# Effect of bone marrow aspiration concentrate and platelet-rich plasma combination in anterior cruciate ligament reconstruction: a randomized, prospective, double-blinded study

**DOI:** 10.1186/s13018-023-04512-y

**Published:** 2024-01-03

**Authors:** Yu-Chuan Lin, Yu-Jen Chen, Tsang-Yu Fan, Pei-Hsi Chou, Cheng-Chang Lu

**Affiliations:** 1 Department of Orthopaedic Surgery, Kaohsiung Medical University Hospital, Kaohsiung, Taiwan; 2 Department of Radiology, Kaohsiung Medical University Hospital, Kaohsiung, Taiwan; 3 School of Medicine, College of Medicine, Kaohsiung Medical University, Kaohsiung, Taiwan; 4 Department of Orthopaedic Surgery, Kaohsiung Municipal Siaogang Hospital, Kaohsiung, Taiwan; 5 Regenerative Medicine and Cell Therapy Research Center, Kaohsiung Medical University, Kaohsiung, Taiwan; 6 Orthopaedic Research Center, Kaohsiung Medical University, Kaohsiung, Taiwan

**Keywords:** Graft healing, Graft maturation, Laxity, MRI

## Abstract

**Background:**

The effect of bone marrow aspirate concentrate (BMAC) and platelet-rich plasma (PRP) combination in enhancing graft maturation and tendon–bone tunnel interfacial healing after anterior cruciate ligament (ACL) reconstruction remains unclear. We hypothesised that BMAC and PRP combination could lead to better clinical results and better graft maturation/interface healing than PRP alone or conventional ACL reconstruction without any other biologic augmentation.

**Methods:**

In this randomised double-blind prospective study, patients undergoing ACL reconstruction surgery were randomly assigned into three groups: (1) control group (without any biologic augmentation), (2) PRP treatment group, and (3) combined BMAC and PRP (BMAC + PRP) group. Moreover, they were evaluated using the clinical functional score, laxity examination, and magnetic resonance imaging (MRI) analysis.

**Results:**

No significant difference was observed in the improvement of functional scores among groups. However, laxity improvement at 24 weeks showed a significant difference with the BMAC + PRP group having the lowest laxity. MRI analysis showed no significant change in whole graft maturation among groups. In particular, the BMAC + PRP group showed delayed signal peak and higher graft signal at 24 weeks compared with the other two groups; however, the difference was not significant. With regard to tendon–bone interfacial healing, the BMAC + PRP group showed significantly wider tendon–bone interface in the femoral bone tunnel at 24 weeks compared with the other two groups. Moreover, the BMAC + PRP group showed significantly higher peri-tunnel edema signal in the femoral bone tunnel at 12 weeks compared with the other two groups.

**Conclusion:**

PRP alone and BMAC and PRP combination showed limited enhancing effect in clinical function, graft maturation and tendon–bone interfacial healing compared with control (no additional treatment). When BMAC is used in ACL reconstruction, the possibility of greater inflammation in the early stage to graft maturation and bone tunnel healing should be considered.

## Introduction

Anterior cruciate ligament (ACL) reconstruction is indicated in patients with persistent pain, weakness, and instability after failed conservative treatment for ACL injuries [1–5]. Factors that affect knee joint stability after ACL reconstruction include implanted graft maturation and interfacial healing between graft and bone tunnel [6–8]. The implanted graft undergoes a maturation process called ligamentisation, comprising three stages: graft necrosis, recellularisation and remodelling [6]. The interfacial healing between graft and bone tunnel requires differentiated interposed cells, which form collagen fibres to stabilise the implanted graft [9]. Without early and timely graft maturation and interfacial healing, the implanted graft undergoes microtear, which leads to graft failure and interfacial loosening. Therefore, regulating the secretion of growth factors and cell repopulation is important to enhance implanted graft maturation and tendon–bone tunnel interface healing after ACL reconstruction to prevent graft retear [10, 11].

Platelet-rich plasma (PRP), harvested from autologous peripheral blood, contains multiple growth factors which promote and regulate the regeneration of damaged tissue by stimulating cell proliferation, migration and differentiation [12, 13]. Accordingly, previous animal and magnetic resonance imaging (MRI) studies showed that PRP improved healing of ACL partial tear, graft maturation after ACL reconstruction, and augmentation in tendon–bone tunnel interface [14–18]. Bone marrow stromal cells (BMSCs), harvested and cultured from bone marrow, are rich in stem cells and progenitor cells with stemness and capability to differentiate into different tissues. These cells have been applied in tendon regeneration and tendon–bone tunnel healing [19–21]. However, the prolonged culture time, high cost, and risk of pathogen contamination and generic alterations limit the clinical application of BMSCs in ACL reconstruction [22]. By contrast, bone marrow aspirate concentrate (BMAC) is harvested from the centrifuged bone marrow to separate bone marrow mononuclear cells from other blood cells. As BMAC contains stem cells without the need of culture expansion and can be harvested in a short period during one time surgery or clinic visit, it has demonstrated positive results in treating tendon injuries, knee osteoarthritis, and intervertebral disc degeneration [23–27]. BMAC can also enhance allograft tendon regeneration and tendon–bone tunnel interfacial healing compared with cultured BMSCs [21, 24].

The combination of growth factors and stem cell therapy is ideal to regulate the proliferation, collagen synthesis, and differentiation of stem cells to treat and enhance injured tissue regeneration [28, 29]. Owing to its synergic effect, the combination of bone marrow cells and PRP provides high possibility to enhance graft maturation and tendon–bone tunnel interfacial healing after ACL reconstruction. In a rabbit ACL reconstruction model, Teng et al. [30] found that the combination of BMSCs and PRP presented more mature tendon–bone tunnel interface with higher failure load compared with PRP only and control treatment. However, the effect of BMAC and PRP combination in enhancing graft maturation and tendon–bone tunnel interfacial healing after ACL reconstruction remains unclear. We hypothesised that the combination of BMAC and PRP would result in better clinical outcomes and better graft maturation/interface healing, as assessed by MRI, compared with PRP alone and conventional treatment without biologic augmentation in patients undergoing ACL reconstruction.

## Materials and methods

### Subjects

This study was approved by the institutional review board of Kaohsiung Medical University Hospital (KMUHIRB-F(I)-20170122; ClinicalTrials.gov Identifier: NCT05191732). The inclusion criteria were as follows: (1) male and female patients aged 20–45 years who had ACL rupture with or without meniscus tear and (2) diagnosis confirmed by MRI examination. The exclusion criteria were as follows: (1) patients with multiple ligament injury of the operated knee; (2) revision ACL reconstruction; (3) severe osteoarthritis, infected arthritis, and rheumatoid arthritis; and (4) coagulopathy or low haemoglobin (< 11 g/dL) and platelet levels (< 150,000/mm^3^).

Thirty subjects met the inclusion criteria and were enrolled in this study. Subjects were randomly assigned to three different groups with 10 subjects in each group: (1) conventional ACL reconstruction without any other biologic augmentation (control group), (2) autologous PRP augmentation (PRP group), and (3) combined BMAC and PRP augmentation (BMAC + PRP group). The method of group randomisation was performed through a random number table using a computer. Two subjects in the PRP group and one subject in the BMAC + PRP group withdrew and/or were lost to follow-up because of work and migration to other countries. Thus, 27 patients completed this study.

The control group included 10 subjects (6 males and 4 females). Four subjects were operated on the right knee, and six on the left knee. The PRP group included eight subjects (6 males and 2 females). Five subjects were operated on the right knee, and three on the left knee. The BMAC + PRP group included nine subjects (5 males and 4 females). Three subjects were operated on the right knee, and six on the left knee. All three groups showed no significant difference in age, body weight, height, and body mass index (Table 1).

**Table 1:** Patient profiles comparison between three groups

	Control	PRP	BMAC + PRP	*p* value
Number	10	8	9	
Gender (M/F)	6/4	6/2	5/4	
Right/left knee	4/6	5/3	3/6	
Age	29.7 ± 9.9	28.4 ± 7.8	27.1 ± 3.9	0.72
Body weight (kg)	71.1 ± 24.9	74.6 ± 15.9	70 ± 9.5	0.210
Body height (cm)	168.9	172 ± 7.6	169 ± 7.7	0.313
Body mass index (BMI)	24.7 ± 4.7	25.0 ± 4.1	24.3 ± 2.8	0.538
Associated meniscus tear	9	8	5	
Graft diameter (mm)	8.25	8.28	8	0.785
Graft length (cm)	8.3	86 ± 4.5	83.5 ± 4.1	0.705
Femoral tunnel diameter (mm)	8.2 ± 0.75	8.05 ± 0.60	8.1 ± 0.6	0.704
Tibial tunnel diameter (mm)	8.35 ± 0.71	8.55 ± 0.55	8.2 ± 0.71	0.695

### Preoperative evaluation

Before ACL reconstruction, informed consent and signed permit were obtained. All subjects were examined using functional score [Lysholm knee score; International Knee Documentation Committee (IKDC) 2000] and laxity examination (KT-1000).

### Anterior cruciate ligament reconstruction procedure

**Fig. 1:**
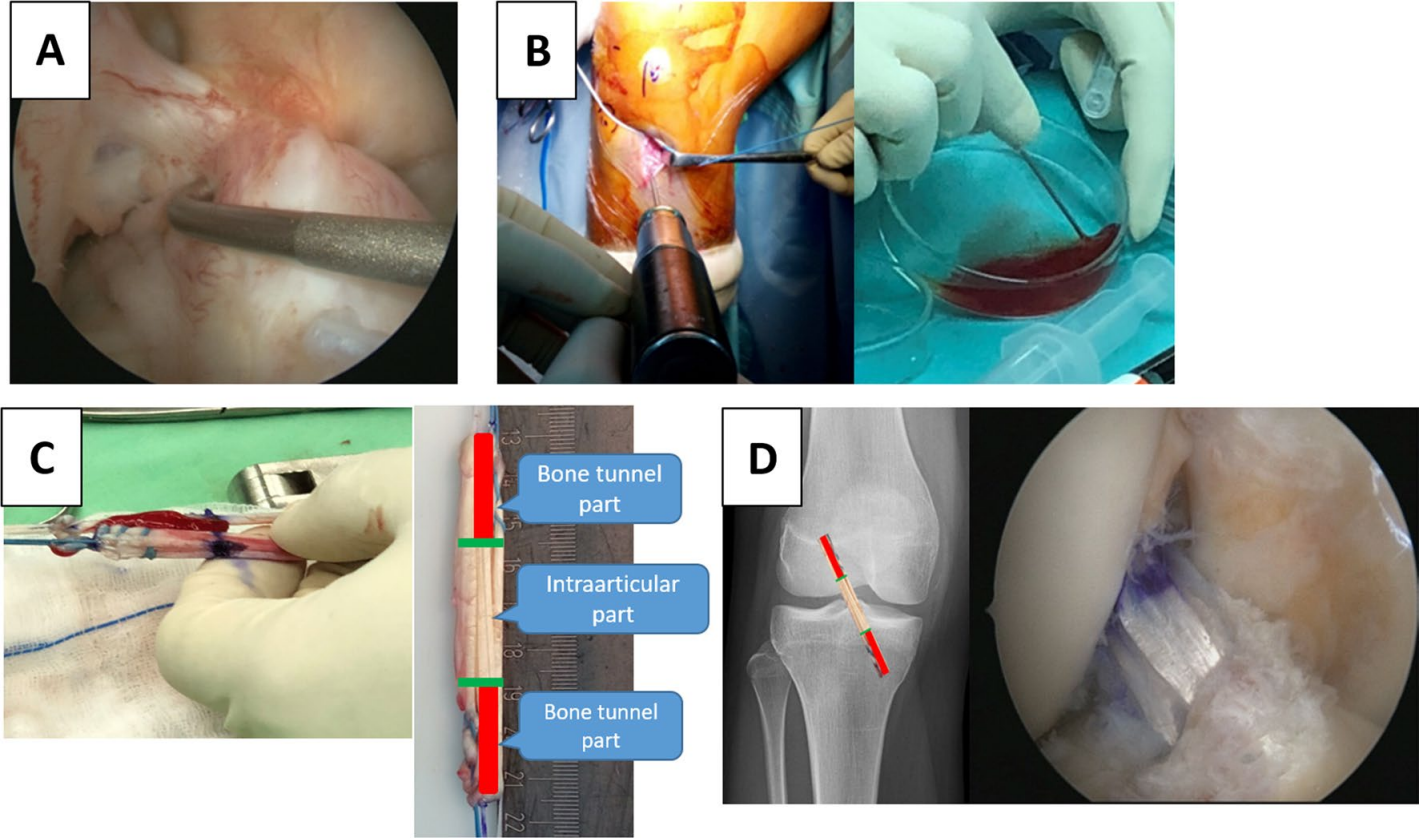
**A** The complete tear of ACL was confirmed under arthroscopy. **B** The bone marrow was harvested from the proximal tibia (hamstring tendon harvest site). After centrifugation, the BMAC was mixed with PRP to form gel. **C** The BMAC + PRP gel were sutured to both ends of graft (bone tunnel parts; marked in red). **D** The prepared graft was passed through the tibial and femoral bone tunnels and fixed with interference screws

Under general anaesthesia, all subjects underwent knee arthroscopic ACL reconstruction with hamstring tendon graft as standard procedure through the tibia to the femur bone tunnel using anatomic single-bundle technique. The procedure was conducted by the senior author (PH Chou). At first, the surgeon confirmed the complete tear of ACL (Fig. 1A). Then, the semitendinosus and gracilis tendons were harvested and prepared to form a fourth (2 semitendinosus/2 gracilis) or fifth-strand (3 semitendinosus/2 gracilis) graft with an average length of 85 mm and width of 7–8 mm according to individual conditions. In the control group, the hamstring graft was prepared without any other biologic augmentation. In the PRP group, 30 ml of peripheral blood (10 ml blood for each tube; commercial PRP tube, Taiwan) was drawn and centrifuged at 3200 rpm for 6 min. Then, PRP from two tubes was mixed and placed on a dish for gel formation. The PRP gel was applied at each end of the graft (bone tunnel side) and fixated using Vicryl 2–0 suture. In the BMAC + PRP group, PRP was harvested as described previously, and 40 ml of bone marrow was collected from the proximal tibia area (hamstring tendon harvest site) using a bone marrow aspiration kit (DBMNI1501, Argon Medical Devices, Athens, TX, USA) with tourniquet deflated. The bone marrow aspirate was injected slowly into the commercial bone marrow cell harvest kit (commercial BMAC tube, Taiwan) and centrifuged at 3600 rpm for 9 min. Then, BMAC and PPR was soaked to form a gel, which was applied at both ends of the graft (Fig. 1B and C).

The tibial tunnel was drilled using a reamer (the same width of the prepared graft) at 50° from the proximal medial tibia to the tibia articular surface centred at the ACL remnant footprint. Using the transtibial technique, the femoral tunnel was created according to the width of the prepared graft to a depth of approximately 30 mm in the 10:30 o’clock (right knee)/01:30 o’clock (left knee) position to the lateral femoral condyle 2 mm anterior to the posterior femoral articular margin with the in-side-out technique. The average graft diameter, graft length, femoral tunnel diameter, and tibia tunnel diameter were compared among groups, and no significant difference was shown (Table 1).

The graft was passed through the tibial and femoral bone tunnels and fixed with interference screws (Smith and Nephew, Andover, MA, USA), which had the same width as the bone tunnel (Fig. 1D). One cancellous post-screw with a washer was applied in the tibia. After wound closure, 3.5 ml of PRP (PRP group) or BMAC + PRP mixture (BMAC + PRP group) was injected into the knee joint. The operated knee was kept on brace and fixed with full extension.

### Postoperative rehabilitation programme

The postoperative rehabilitation programme was conducted by an experienced trainer who was blinded to the patient subgroups. The training course was uniform for all subjects to avoid rehabilitation biases.

Every subject underwent the programme at 1, 3, 5, 7, and 9 weeks post-surgery. The programme was conducted five times (1 h each time with the same trainer). The trainer provided the current training programme and take-home training programme. In the first and third week, the training programme focused on quadriceps muscle isometric contraction, passive and active knee range of motion, hip stability training, and crutch walking training. In the fifth and seventh week, the training programme focused on single/double-leg balance training and closed-chain (i.e. squat, lunge) and open-chain (resistance training with elastic band during knee extension and flexion) lower leg muscle strengthening. In the ninth week, the training course aimed to restore daily activities such as climbing up and down the stairs, standing up, sitting on a chair, and single-leg standing.

### Postoperative evaluation and follow-up

All subjects were evaluated using the functional score, knee laxity, and MRI examination.

#### Functional score (Lysholm knee score and IKDC 2000; pre-surgery, 12, 24, and 48 weeks post-surgery)

The subjects were evaluated for functional outcomes using the Lysholm and IKDC scores before surgery and 12, 24 and 48 weeks after surgery. The Lysholm knee score includes eight items to evaluate subject’s knee condition including pain (25 points), instability (25 points), locking (15 points), swelling (10 points), limp (5 points), stair climbing (10 points), squatting (5 points) and need for support (5 points). Higher scores indicated better outcomes [31]. The IKDC form involves four main areas: subjective assessment, symptoms, range of motion, and ligament examination. Higher scores indicate higher level of knee function with lower level of symptoms [32].

#### Knee laxity examination (KT1000; pre-surgery, 12, 24, and 48 weeks post-surgery)

Knee ligament laxity examination was performed using the KT-1000 arthrometer (Medmetric, Inc., San Diego, CA, USA) before surgery and 12, 24 and 48 weeks after surgery. The examination was performed with the subject lying down and his/her knees flexed at 30°. The examination was repeated three times in the operated and normal knees. The result was recorded as the average of three tests.

#### MRI examination (6, 12, 24 and 48 weeks post-surgery)

MRI examination was performed using a 1.5 Tesla whole-body scanner (Achieva, Philips Healthcare, The Netherlands) and dedicated 8-channel knee coils. Postoperative MRI examination was performed at 6, 12, 24 and 48 weeks after ACL reconstruction. Proton-density-weighted, T1-weighted, and T2-weighted sequences were obtained.

The (1) maturation of implanted graft (graft signal change from T2 sagittal view) and (2) graft tendon–bone tunnel interfacial healing (diameter change of bone tunnel from T1 transverse view; peri-tunnel edema from T2 transverse view) were investigated from the MRI examination. Radiologists who were blinded to the grouping performed all measurements using a conventional PACS system (Sectra Medical Systems, Sweden).

##### Maturation of implanted graft

**Fig. 2:**
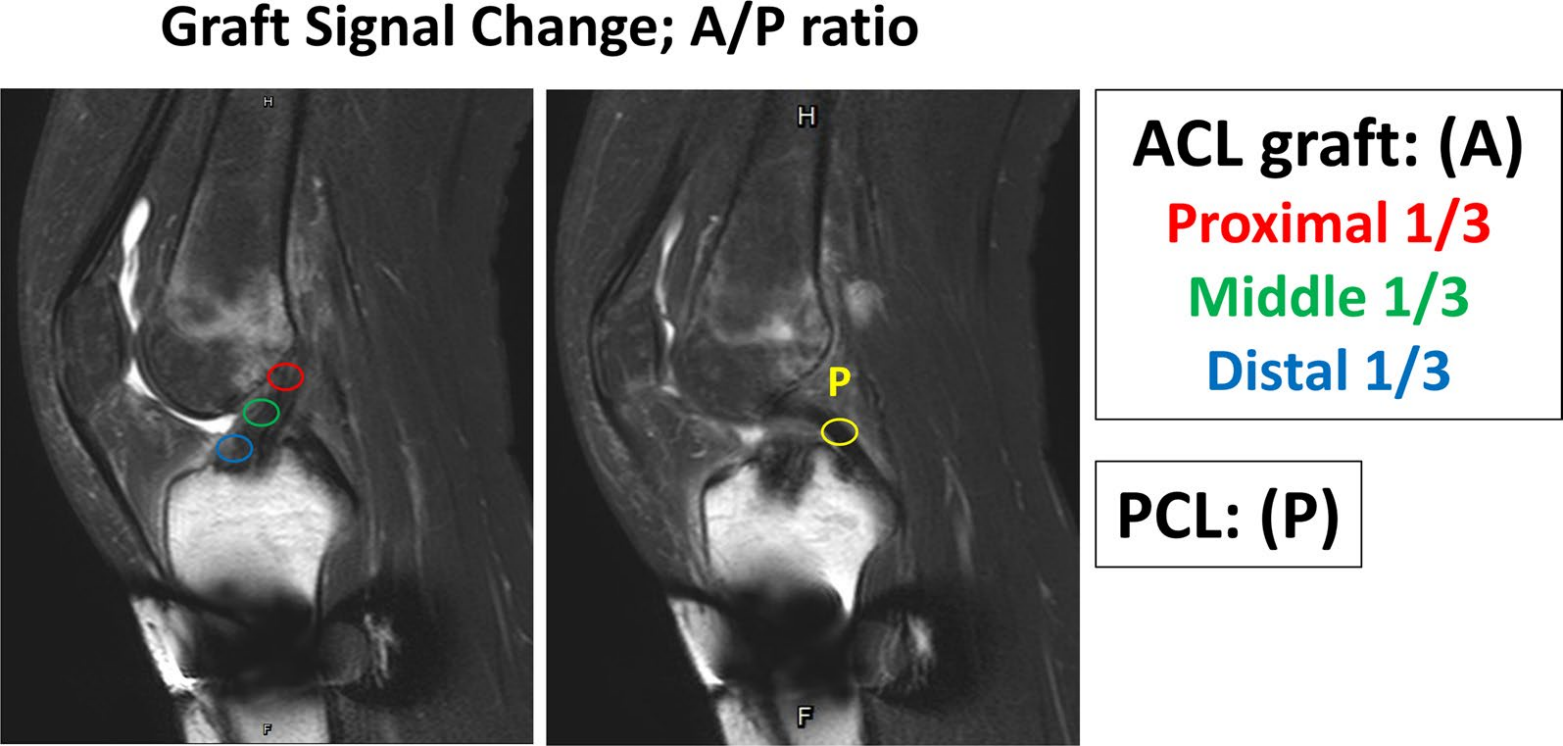
The graft maturation was recorded as graft signal change detected from the T2 sagittal view in MRI. The whole graft signal (proximal, middle, and distal portions of the ACL graft) was averaged and normalised by individual PCL signal (A/P ratio)

T2-weighted sagittal MR images parallel to ACL were used for analysis to investigate the maturation of the implanted hamstring tendon graft with time changes (repetition time 2090 ms, echo time 60 m, field of view 160 mm, in plane resolution 0.33 × 0.33 mm, slice thickness 4 mm, acquisition time 1:48). The MRI signal intensities were measured manually in four regions of interest (ROIs): intraarticular proximal/middle/distal portion of the ACL graft and mid-portion of posterior cruciate ligament (PCL, reference signal). A standardised 4-mm-diameter circle was used for each ROI. The whole graft signal (proximal, middle, and distal portions of the ACL graft) was averaged and normalised by individual PCL signal (A/P ratio) (Fig. 2).

##### Graft tendon–bone tunnel interfacial healing

**Fig. 3:**
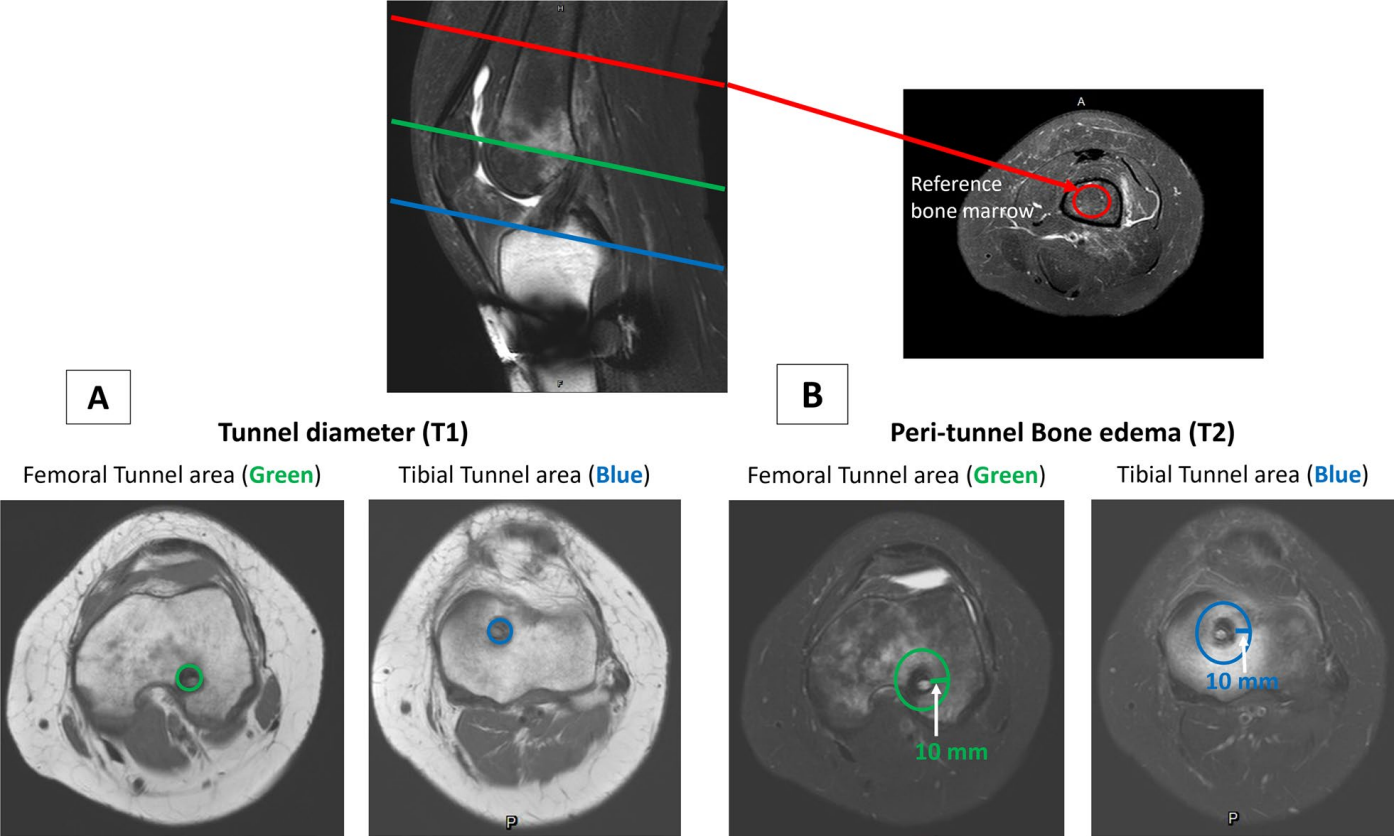
The graft tendon-bone tunnel interfacial healing was measured as the bone tunnel diameter (T1; **A**) and peri-tunnel edema (T2; **B**) at the level of entry of femoral (green) and tibial (blue) bone tunnels. The T2 signal of femoral bone marrow in the middle third of femoral shaft (red) was set as the reference to normalise the individual peri-tunnel edema change

The femoral and tibial bone tunnel diameter was measured at the level of graft entry from T1 transverse view to investigate implanted graft tendon–bone tunnel healing (Fig. 3A). The serial change of bone tunnel diameter was calculated by dividing the measured bone tunnel diameter with the original bone tunnel diameter. In addition, peri-tunnel edema was observed from T2 transverse view by measuring the signal from the 10 mm circle area to the centre of the bone tunnel. The signal of bone marrow in the middle third of the femoral shaft was set as the reference point (Fig. 3B). The change of peri-tunnel edema was recorded by dividing the measured peri-tunnel T2 signal with the reference bone marrow signal.

### Statistical analysis

One-way analysis of variance (ANOVA) and post-hoc analysis with the Kruskal–Wallis test were performed to determine the difference of functional score, knee laxity, and MRI result at each examination time point among the control, PRP, and BMAC + PRP groups. Paired t-test was used to analyse the change at each examination time point in each group. Statistical significance was considered at *p* < 0.05. All statistical analyses were conducted using SPSS version 20.0 for Windows (SPSS Inc. Chicago, IL, USA). Data are presented as the mean ± standard deviation.

## Results

### Functional score analysis

#### *All groups together (*Fig. 4A*)*

**Fig. 4:**
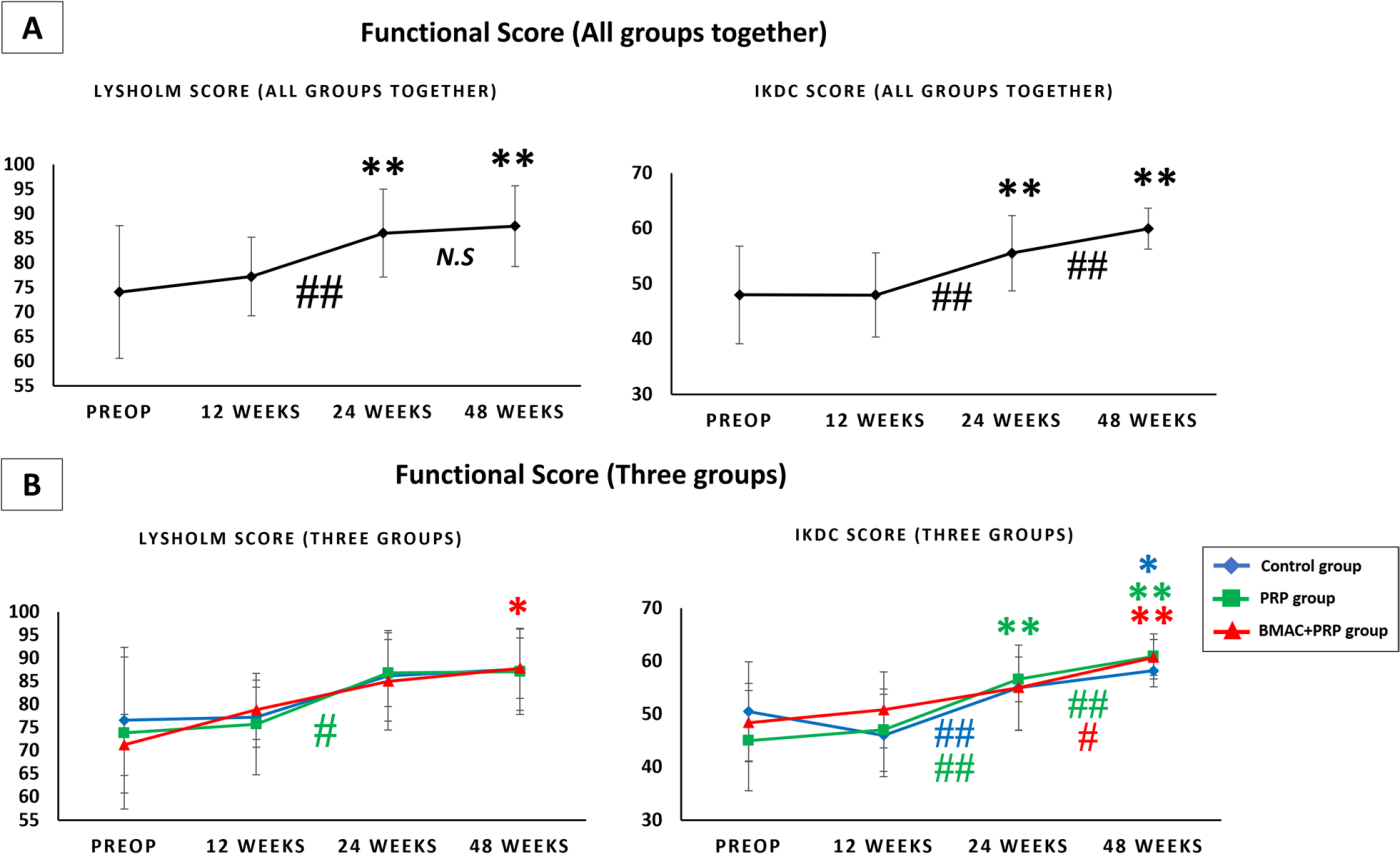
**A** Functional score (Lyholsm score and IKDC score) in all groups together. **B** Functional score (Lyholsm score and IKDC score) in control group, PRP group and BMAC + PRP group. *p < 0.05 versus preop; **p < 0.01 versus preop; #*p* < 0.05 between weeks; ##*p* < 0.01 between weeks; N.S: no significant difference between weeks

In all groups together, the functional score increased with time. A significant increase in the Lysholm and IKDC scores was observed between 12 and 24 weeks; however, only the IKDC score showed a significant increase between 24 and 48 weeks.

#### *Three groups (*Fig. 4B*)*

The Lysholm and IKDC scores improved with time in all three groups. No significant difference was observed in the functional score at different time points among all groups.

### Knee laxity analysis

#### *All groups together (*Fig. 5A*)*

**Fig. 5:**
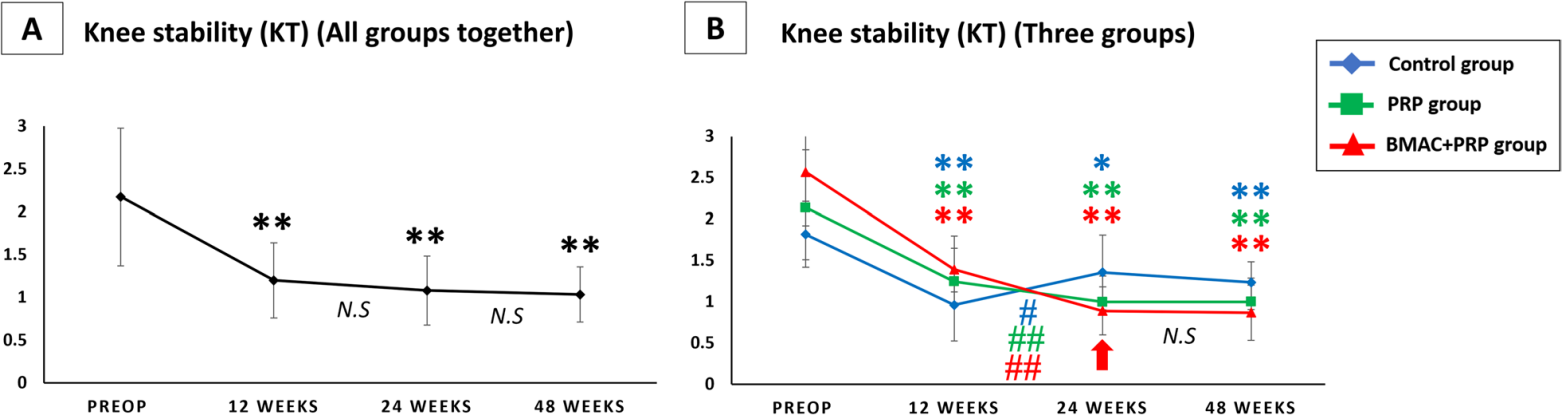
**A** Knee laxity result (KT 1000) in all groups together. **B** Knee laxity result (KT 1000) in control group, PRP group and BMAC + PRP group. The red arrow indicates significant difference between three groups by one way ANOVA. *p < 0.05 versus preop; **p < 0.01 versus preop; #*p* < 0.05 between weeks; ##*p* < 0.01 between weeks; N.S: no significant difference between weeks

In all groups together, knee laxity was significantly improved at 12, 24 and 48 weeks after operation compared with that before operation. However, no significant difference was found between 12–24 weeks and 24–48 weeks, indicating that laxity in all groups together improved and remained unchanged at 12 weeks after ACL reconstruction.

#### *Three groups (*Fig. 5B*)*

Knee laxity decreased gradually at 12 weeks after operation, and the difference was significant. From 12 to 24 weeks post-ACL reconstruction, the control group had significantly increased laxity, whereas the PRP and BMAC + PRP groups had significantly decreased laxity. However, the BMAC + PRP group had significantly improved laxity at 24 weeks compared with the control group, and the difference was significant (*p* = 0.029).

### MRI factor analysis

#### Graft maturation

##### Graft maturation signal change in all groups together (Fig. 6A)

**Fig. 6:**
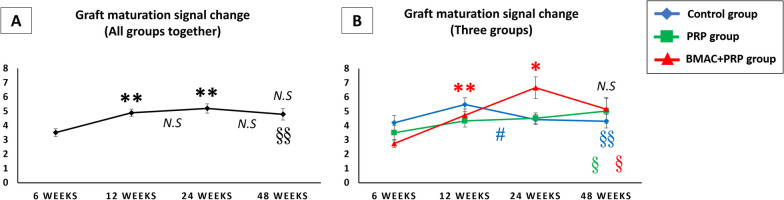
**A** Graft maturation signal change (A/P ratio) in all groups together. **B** Graft maturation signal change (A/P ratio) in control group, PRP group and BMAC + PRP group. *p < 0.05 versus 6 weeks; **p < 0.01 versus 6 weeks; #*p* < 0.05 between weeks; N.S: no significant difference versus 6 weeks or between weeks; §*p* < 0.05 versus PCL signal (reference); §§*p* < 0.01 versus PCL signal (reference)

In all groups together, the graft signal increased gradually at 12 and 24 weeks after operation, peaking at 24 weeks and then decreasing. At 48 weeks, the graft signal was still significantly higher than the PCL signal.

##### Graft maturation signal change in three groups (Fig. 6B)

In the control group, the graft signal peaked at 12 weeks and then significantly decreased at 12 to 24 weeks. In the PRP group, the graft signal did not significantly change from 6 to 48 weeks. In the BMAC + PRP group, the graft signal significantly increased and peaked at 24 weeks and then decreased. The graft signal peak occurred later in the BMAC + PRP group (24 weeks) than in the control group (12 weeks). At 48 weeks after ACL reconstruction, the graft signal in all three groups were still significantly higher than that of PCL (reference). At 24 weeks, the graft signal in the BMAC + PRP group was higher than that of the control and PRP groups; however, the difference was not significant.

#### Tendon–bone tunnel healing

##### Bone tunnel diameter change in all groups together (Fig. 7A)

**Fig. 7:**
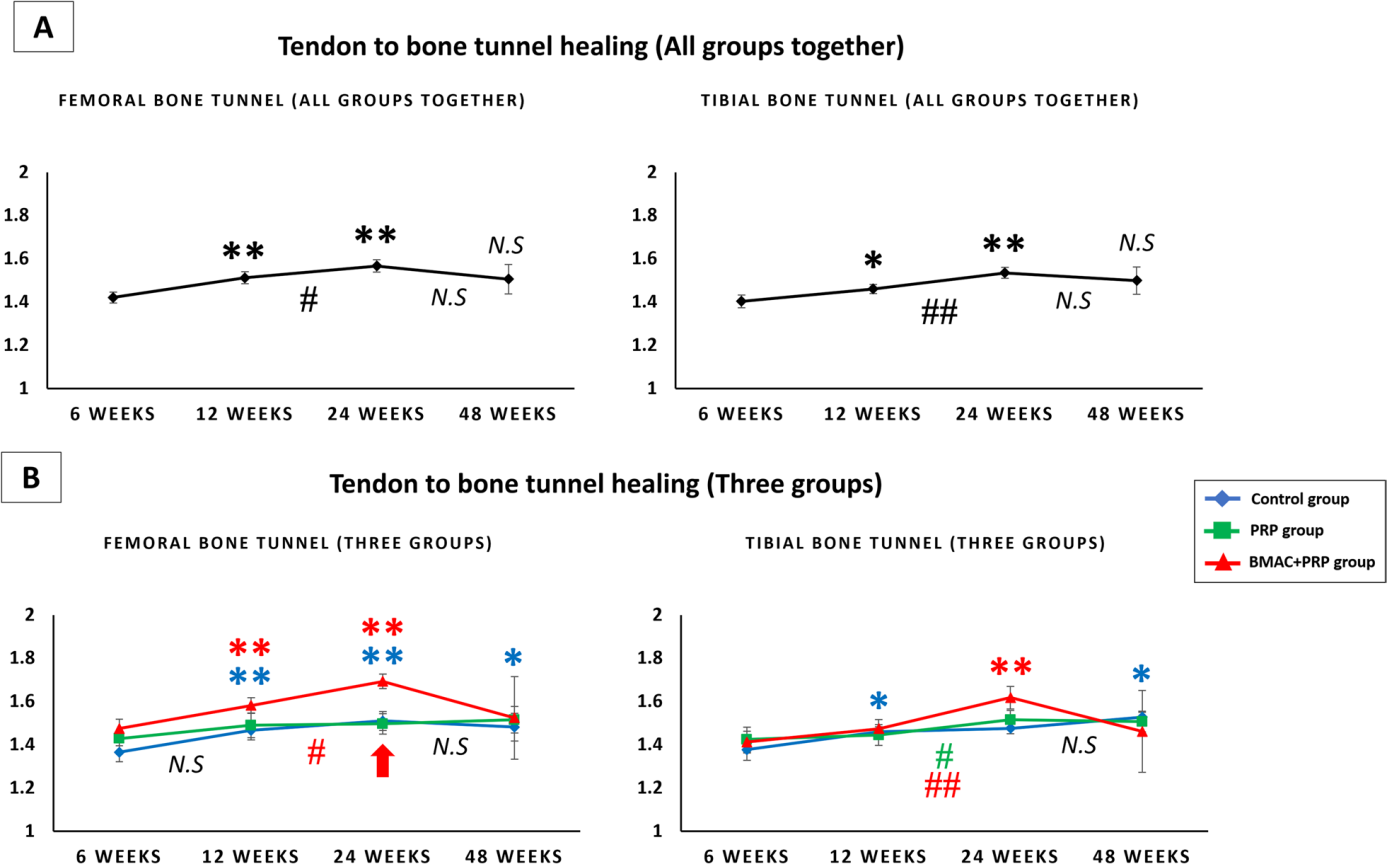
**A** The femoral and tibial bone tunnel diameter change in all groups together. **B** The femoral and tibial bone tunnel diameter change in control group, PRP group and BMAC + PRP group. The red arrow indicates significant difference between three groups by one way ANOVA. *p < 0.05 versus 6 weeks; **p < 0.01 versus 6 weeks; #*p* < 0.05 between weeks; ##*p* < 0.01 between weeks; N.S: no significant difference versus 6 weeks or between weeks

In all groups together, the femoral and tibial bone tunnel diameter increased significantly at 12 and 24 weeks, peaking at 24 weeks and then decreasing. The femoral and tibial bone tunnel diameter was wider at 48 weeks than at 6 weeks; however, the difference was not significant. In particular, the femoral and tibial bone tunnel diameter significantly increased at 12–24 weeks.

##### Bone tunnel diameter change in three groups (Fig. 7B)

In the control group, the femoral bone tunnel significantly increased at 12, 24, and 48 weeks, whereas the tibial bone tunnel increased at 12 and 48 weeks. In the PRP group, the femoral and tibial bone tunnel did not change significantly with time. However, the tibial bone tunnel significantly increased at 24 weeks than that at 12 weeks. In the BMAC + PRP group, the femoral bone tunnel significantly increased at 12 and 24 weeks than that at 6 weeks, whereas the tibial bone tunnel significantly increased at 24 weeks than that at 6 weeks. However, they decreased at 48 weeks. At the same time, in the BMAC + PRP group, there was significantly increase at 24 weeks than that at 12 weeks in both femoral and tibial bone tunnel.

In comparing the three groups, the femoral bone tunnel diameter showed a significant difference at 24 weeks (control vs. BMAC + PRP, *p* = 0.024; PRP vs. BMAC + PRP, *p* = 0.008), with the BMAC + PRP group having the widest diameter.

#### Peri-bone tunnel edema

##### Peri-tunnel edema in all groups together (Fig. 8A)

**Fig. 8:**
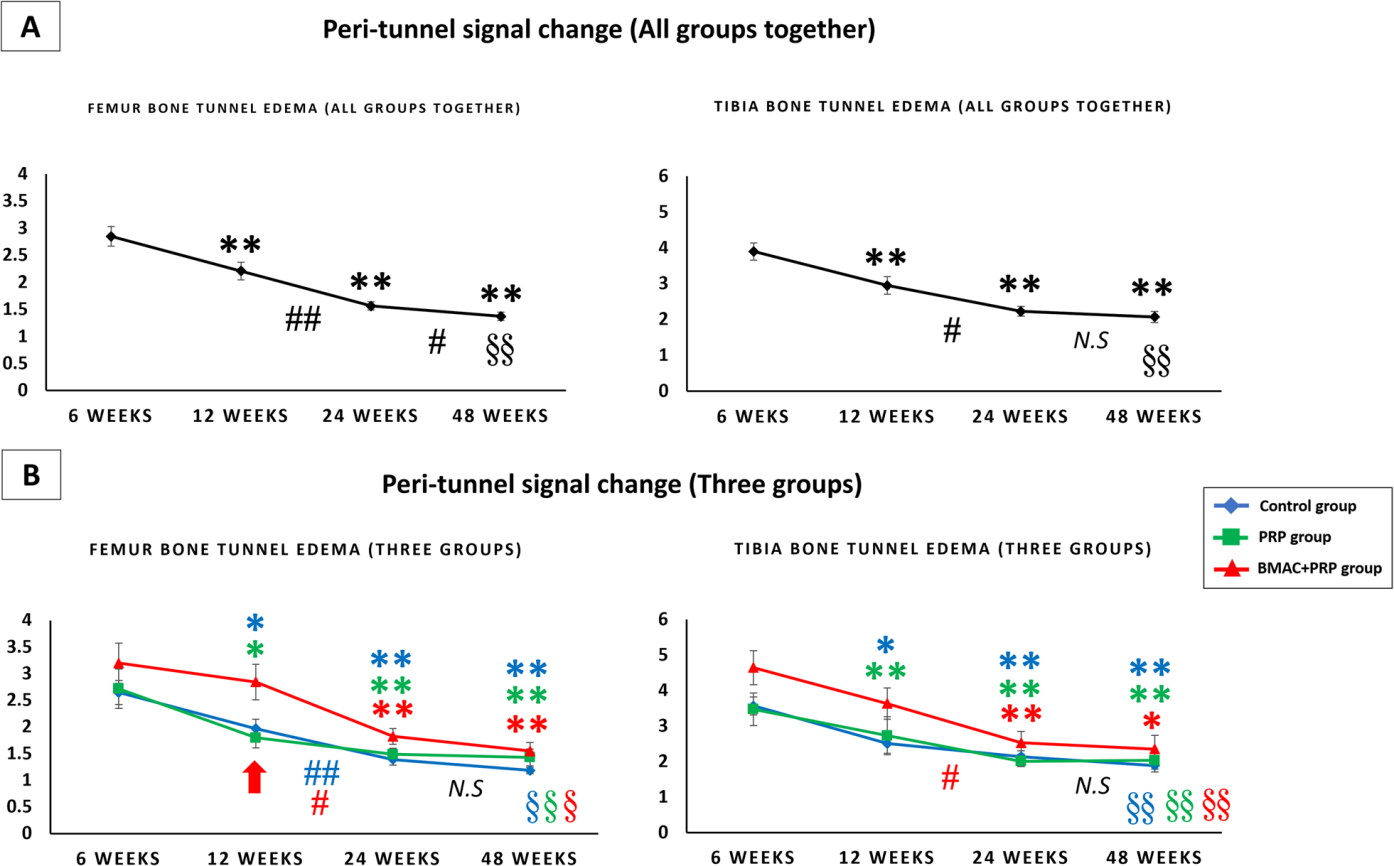
**A** The peri-tunnel bone marrow signal change in femoral and tibial bone in all groups together. **B** The peri-tunnel bone marrow signal change in femoral and tibial bone in control group, PRP group and BMAC + PRP group. The red arrow indicates significant difference between three groups by one way ANOVA. *p < 0.05 versus 6 weeks; **p < 0.01 versus 6 weeks; #*p* < 0.05 between weeks; ##*p* < 0.01 between weeks; N.S: no significant difference between weeks; §*p* < 0.05 vs reference femur bone marrow signal; §§ *p* < 0.01 versus reference femur bone marrow signal

Peri-tunnel edema in the femoral and tibial bone tunnel significantly decreased at 12, 24 and 48 weeks compared with that at 6 weeks. At 48 weeks after ACL reconstruction, a significantly higher signal was found around the peri-tunnel area compared with the reference femoral bone marrow signal, indicating that peri-tunnel edema was still present at 48 weeks after operation.

##### Peri-tunnel edema in three groups (Fig. 8B)

In all three groups, the highest edema signal was observed at 6 weeks, which gradually decreased with time. In the BMAC + PRP group, no significant difference was observed in the femoral and tibial bone tunnel at 12 weeks compared with that at 6 weeks. However, the signal significantly decreased at 12–24 weeks.

In comparing the three groups, a significant difference was found in the femoral bone tunnel at 12 weeks, with the BMAC + PRP group having the highest edema signal (PRP vs. BMAC + PRP, *p* = 0.17). At 48 weeks after ACL reconstruction, peri-bone tunnel edema was still significantly higher than the reference bone marrow area in all three groups.

## Discussion

Enhancing graft maturation and tendon–bone tunnel healing is important after ACL reconstruction. In this study, PRP or BMAC + PRP gel was applied at both ends of the tendon graft, and then PRP solution or BMAC + PRP mixture was intraarticularly injected. The results were evaluated using the clinical functional score, laxity and MRI examination. No significant difference was observed in functional score improvement among the three groups. There was a significant difference in laxity improvement at 24 weeks, with the BMAC + PRP group showing the best knee stability among the three groups. MRI showed no significant change in whole graft maturation among the three groups. In particular, the BMAC + PRP group showed delayed signal peak and higher graft signal at 24 weeks compared with the other two groups; however, the difference was not significant. With regard to tendon–bone interfacial healing, the BMAC + PRP group had significantly wider tendon–bone interface in the femoral bone tunnel at 24 weeks compared with other groups. Peri-tunnel edema showed significantly higher signal in the femoral bone tunnel of the BMAC + PRP group at 12 weeks compared with those of the PRP and control groups. The overall study indicated the limited enhancing effect in clinical function, graft maturation and tendon–bone interfacial healing, with only significant knee laxity improvement in the BMAC + PRP group at 24 weeks compared with the control group (no biologic augmentation).

For all groups, the functional score increased gradually with time after ACL reconstruction. In particular, the Lysholm and IKDC scores significantly increased between 12 and 24 weeks. At the same time, knee stability improved gradually. Significant improvement was found during the first 12 weeks, and no significant changes were observed at 24 and 48 weeks. Analysis of MRI results in all groups showed that the graft signal gradually increased along with the bone tunnel until 24 weeks and then decreased. Peri-tunnel bone edema decreased progressively with time. At 48 weeks after ACL reconstruction, the graft showed significantly higher signal than the PCL and wider femoral and tibial bone tunnel than that at 6 weeks. Moreover, the peri-tunnel bone marrow signal did not return to normal. These results indicated that the function and stability of the knee improved progressively with time. However, graft maturation and tendon–bone interfacial healing was not completed even after 48 weeks of ACL reconstruction in all three groups.

The effect of PRP application in ACL reconstruction is still debated. Its ability to reduce postoperative pain and improve ACL graft maturation has been reported. However, adding PRP alone did not show benefits in improving clinical knee score or knee stability, reducing bone tunnel widening or accelerating tendon–bone healing compared with conventional ACL reconstruction [14, 33, 34]. In their prospective randomised controlled study, Gong et al. [14] introduced investigated the effect of PRP application in graft maturation and tendon–bone tunnel healing after ACL reconstruction. Based on the results of computed tomography and MRI examination, they found that the intraarticular graft signal and bone tunnel diameter widening did not show any significant difference between the PRP application group and the conventional group. In the present study, no significant difference was observed in the functional score, knee laxity and MRI results after PRP application compared with control group after ACL reconstruction.

The combination of PRP and cell therapy has been proposed to enhance ligament/tendon regeneration and tendon–bone interfacial healing in clinical and animal studies [19, 21, 28, 29]. In 2013, Martin et al. [35] used BMAC and PRP combination to treat femoral head osteonecrosis during decompression procedure. Their results showed significant pain relief in 86% of patients without major complications. Centeno et al. [25] used BMAC and PRP combination with direct injection to the partial ACL area with minimal retraction in 29 patients. They found that 77% of patients had improved ACL injury. The combination of BMAC and PRP also accelerated bone healing in patients with long bone nonunion [36]. Reasonably, the combination of BMAC and PRP would present a positive effect to enhance the healing of intraarticular tendon graft and tendon–bone tunnel compared with conventional ACL reconstruction. However, in this study, BMAC + PRP combination only showed a limited positive effect in improving knee laxity at 24 weeks after operation compared with the control. Moreover, it showed no significant benefit in improving functional scores at all time points compared with the PRP and control groups. At the same time, the BMAC + PRP group presented higher intraarticular graft signal, enlarged tendon–bone interface, and higher peri-tunnel bone marrow edema signal compared with the control and PRP groups.

To the best of our knowledge, no studies have directly compared the enhancing effect of PRP and BMAC and PRP combination in ACL reconstruction using a prospective, randomised, and double-blind method. In the present randomised double-blind prospective study, fresh bone marrow was harvested from the hamstring graft harvest site during ACL reconstruction, enabling the blinding of patients without other harvest sites. In addition, we combined the clinical study (functional score, knee laxity) with serial MRI examination to investigate the effect of PRP and BMAC + PRP on graft maturation and tendon–bone tunnel interfacial healing. However, this study had limitations. We did not perform element evaluation of PRP and BMAC, therefore, the composition of the aspirated substances was not certain and was not under control. In this study, bone marrow was aspirated from the proximal tibia. Possible differences of bone marrow activity from the iliac crest or other donor sites remain unknown. Although the surgical procedures were performed by the same surgeon using the same technique, but it is difficult to always place the grafts in the same position and with the same tension. The sample size in each group was small, and the follow-up time was short (48 weeks).

## Conclusion

The overall study indicated that PRP and BMAC + PRP had limited enhancing effect in clinical function, graft maturation, and tendon–bone interfacial healing compared with conventional treatment. When BMAC is used in ACL reconstruction, the possibility of greater inflammation in the early stage to graft maturation and bone tunnel healing should be considered.
